# The influence of probe level on the tuning of stimulus frequency otoacoustic emissions and behavioral test in human

**DOI:** 10.1186/s12938-016-0167-0

**Published:** 2016-05-10

**Authors:** Yao Wang, Qin Gong, Tao Zhang

**Affiliations:** Department of Biomedical Engineering, School of Medicine, Tsinghua University, Beijing, 100084 China; Research Center of Biomedical Engineering, Graduate School at Shenzhen, Tsinghua University, Shenzhen, 518055 China; Tsinghua National Laboratory for Information Science and Technology (TNList), Tsinghua University, Beijing, 100084 China

**Keywords:** Frequency selectivity (FS), Stimulus frequency otoacoustic emission suppression tuning curves (SFOAE STCs), Psychophysical tuning curves (PTCs), Probe level

## Abstract

**Background:**

Frequency selectivity (FS) of the auditory system is established at the level of the cochlea and it is important for the perception of complex sounds. Although direct measurements of cochlear FS require surgical preparation, it can also be estimated with the measurements of otoacoustic emissions or behavioral tests, including stimulus frequency otoacoustic emission suppression tuning curves (SFOAE STCs) or psychophysical tuning curves (PTCs). These two methods result in similar estimates of FS at low probe levels. As the compressive nonlinearity of cochlea is strongly dependent on the stimulus intensity, the sharpness of tuning curves which is relevant to the cochlear nonlinearity will change as a function of probe level. The present study aims to investigate the influence of different probe levels on the relationship between SFOAE STCs and PTCs.

**Methods:**

The study included 15 young subjects with normal hearing. SFOAE STCs and PTCs were recorded at low and moderate probe levels for frequencies centred at 1, 2, and 4 kHz. The ratio or the difference of the characteristic parameters between the two methods was calculated at each probe level. The effect of probe level on the ratio or the difference between the parameters of SFOAE STCs and PTCs was then statistically analysed.

**Results:**

The tuning of SFOAE STCs was significantly positively correlated with the tuning of the PTCs at both low and moderate probe levels; yet, at the moderate probe level, the SFOAE STCs were consistently broader than the PTCs. The mean ratio of sharpness of tuning at low probe levels was constantly around 1 while around 1.5 at moderate probe levels.

**Conclusions:**

Probe level had a significant effect on the sharpness of tuning between the two methods of estimating FS. SFOAE STC seems a good alternative measurement of PTC for FS assessment at low probe levels. At moderate probe levels, SFOAE STC and PTC were not equivalent measures of the FS in terms of their bandwidths. Because SFOAE STCs are not biased by higher levels auditory processing, they may represent cochlear FS better than PTCs.

## Background

Frequency selectivity (FS), an ability of decomposing frequency components of a complex stimulus, plays a crucial role in the auditory perception [1]. It can be assessed noninvasively in humans by measurements of psychophysical tuning curves (PTCs), where the masked threshold for a fixed tone is tracked across a range of masker frequencies [2–4]. To avoid the influence of beat detection, a narrow band noise rather than a tone is adopted as a masker in PTCs [3, 5]. Although PTC can evaluate FS quickly [6–8], its utilization is hampered within non-responsive populations by its subjectivity and strong dependence on subjects’ cooperation. Despite such shortcomings, PTCs have frequently been used to assess FS in patients with sensorineural hearing impairment [9–11]. And the sharpness of PTCs measured in the patients with cochlear hearing loss are usually indicating worsened FS [12]. In order to compare the FS in normal-hearing and hearing-impaired subjects, PTCs at higher probe levels are generally necessary. Thus, many researchers investigated the effect of increasing probe level on the sharpness of PTCs in normal-hearing subjects, but the results seemed inconsistent. Broader tuning of PTCs was observed at higher probe levels in some investigations [13–15]. However, Stelmachowcz et al. reported that the tuning of PTCs characterized by *Q*_10_, which was the ratio of the tip frequency (*f*_tip_) and the bandwidth at 10 dB above the tuning curve tip (BW10), increased with probe levels ranging from 20 to 50 dB sound pressure level (SPL) and then kept stably over 50 dB SPL [16]. Regardless of the uncertainty of the tendency for PTCs’ tuning at higher probe levels, PTCs’ subjectivity remains a limitation for clinical use and an objective method for FS estimate is required.

Stimulus frequency otoacoustic emission (SFOAE) is an acoustic emission evoked by a tone at a single frequency, and generally considered as the result of activities of the cochlear mechano-electrical transducer [17]. It can be evoked within wide range of frequencies for subjects with normal hearing or moderate hearing impairment [18]. In 1980, Kemp et al. found that fully suppressed SFOAE suppression tuning curve (STC) matched closely the tone-on-tone total masking PTC, and firstly predicted that SFOAE STCs might be used to evaluate cochlear function objectively [19]. Since their pioneering work, despite the complexity of SFOAE extraction [20–22] and uncertainty about the mechanism of SFOAE generation [23–25], SFOAE STCs are regarded as reflecting the auditory tuning in some laboratory species [26, 27] and behavioural tuning in humans [28–30]. Additionally, the effect of probe level on the tuning of SFOAE STCs and other types of measurement for FS based on SFOAEs (e.g., SFOAE group delays) were observed in many investigations [27–29, 31]. Keefe et al. measured SFOAE STCs in humans at probe levels of 20–60 dB SPL and reported that the quality factor based on the equivalent rectangular bandwidth (*Q*_ERB_, indicating the tuning sharpness) of SFOAE STCs varied little with probe level changing [28]. However, Charaziak et al. showed that the sharpness of SFOAE STCs decreased with increasing probe level from 10 to 30 dB sensation level (SL) at 4 kHz, but not at 1 kHz in humans [29]. The tuning predicted from SFOAE group delay decreasing with increasing probe level from 40 to 70 dB SPL in humans was reported by Schairer et al. [31]. For laboratory species, Cheatham et al. reported that SFOAE STCs in wild-type mice might get broader at moderate probe levels [26]. The decreasing in sharpness of SFOAE STCs tuning with increasing probe level was also observed in chinchillas, but the *Q*_10_ derived from SFOAEs group delay in chinchillas revealed no significant dependence on probe levels [27]. In summary, the effect of increasing probe level on the tuning of SFOAE STCs or other FS measurements based on SFOAEs appears controversial.

After the first proposal of Kemp and Chum [19] that SFOAE STCs might be applied objectively to estimate FS, SFOAEs were showed to have similar tuning to human psychophysical measurements at low probe levels [28–30]. However, higher probe levels may lead to the saturation and nonlinear compression of the basilar membrane (BM), which is relevant to the FS at the cochlear level [32–34]. Therefore, how the tuning relationship between SFOAE STCs and PTCs varies with probe levels remains a question. To the best of our knowledge, although there were several studies about the tuning variation of SFOAE STCs or PTCs with increasing probe level, little investigation was published about the effect of the probe level on the tuning relationship for the FS estimation between the cochlear level (SFOAE STCs) and the behavioural level (PTCs). The aim of the present study is to investigate the influence of the probe level on the tuning of SFOAE STCs and PTCs. First, SFOAE STCs and PTCs were collected in normal-hearing subjects for frequencies centred at 1, 2, and 4 kHz at both low and moderate probe levels. Then, various parameters (*Q*_10_, slopes, frequency shift and level at the tip) relevant to interpreting the shape and tuning characteristics were calculated for each method at different probe levels. Furthermore, the statistical analysis was conducted to determine the effect of the probe level on the relationship between the parameters of SFOAE STCs and PTCs.

## Methods

### Subjects

Fifteen young subjects (10 females, 5 males) aged from 20 to 30 years old (mean ± standard deviation: 22 ± 3.26) participated in this study. Data were collected in 9 left ears and 6 right ears; the tested ear was randomly determined. All subjects were native Chinese speakers from Tsinghua University with normal hearing (<15 dB hearing level for octave frequencies of 250–8000 Hz). None had a history of hearing disorders or spontaneous otoacoustic emissions (SOAEs), which will interact with the SFOAE extraction [35] and PTC measurements [36], in ± 300 Hz around the probe frequency. All subjects were given their written informed consent to participate and paid for their participation, in compliance with a protocol approved by the institutional review board at Tsinghua University (IRB00008273).

### Instrumentation

All experiments were carried out with subjects comfortably sitting on a chair in a sound-attenuating booth, using the same instruments as described in detail in our previous paper [30]. In brief, stimuli were generated by an external soundcard (Fire face 800, RME, Haimhausen, German) and delivered to subjects by inserted earphones (ER-2, Etymotic Research, Elk Grove Village, IL, USA). Acoustic signals collected in the ear canal by a miniature microphone (ER-10B+ , Etymotic Research, Elk Grove Village, IL, USA) with an amplification of 20 dB (ER-10B+ preamplifier, Etymotic Research, Elk Grove Village, IL, USA) were recorded by the soundcard. A monaural earplug which contained both earphones and microphone was inserted into the ear canal of the subject using a soft ear tip. In the detection of SFOAE STCs, low-frequency background noise was removed by a zero phase shift high-pass filter (cut-off frequency at 500 Hz). In the detection of PTCs, each subject was instructed to pay attention to the probe tone by pressing/releasing a USB handle button when the tone can/cannot be heard. The measurement system was calibrated with a Brüel and Kiær ear simulator type 4157.

### Data collection

**Fig. 1:**
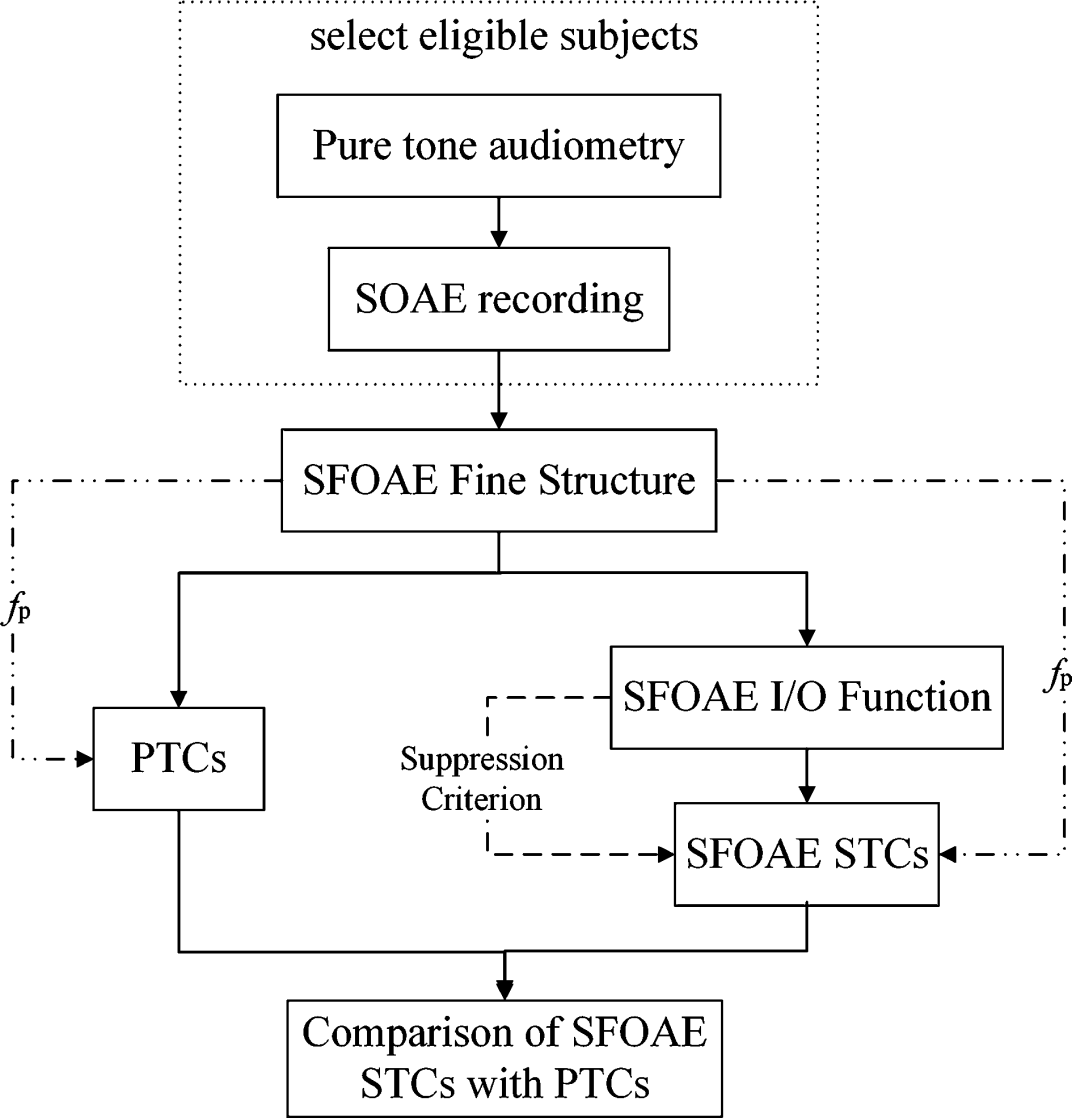
A flowchart for experiment procedure. Flowchart of the experiment procedure

Data were collected within 6 hours (in three separate sections) for each subject at both low and moderate probe levels at 1, 2, and 4 kHz. Singular SFOAE STC recording lasted ~30 min for a frequency resolution of 10 points per octave and singular PTC recording lasted ~8 min. As shown in Fig. 1, the pure tone audiometry and SOAE recording were conducted firstly for subject selection. Then a high-resolution (40 Hz steps) scanning of SFOAE levels (represents the SFOAE fine structure) was performed to determine *f*_*p*_, which is the frequency that can evoke the largest SFOAE within ± 200 Hz relative to the nominal frequencies of 1, 2, and 4 kHz. The suppressor frequency (*f*_s_) was 47 Hz below the *f*_*p*_ ensuring that each *f*_s_ was not the harmonic or subharmonic of the *f*_*p*_. The suppressor level (*L*_s_) was 70 dB SPL in SFOAE fine structure and SFOAE input/output (I/O) function test subsequently. SFOAE I/O function, defined as the SFOAE levels as a function of *L*_*p*_ (5–50 dB SPL in 5 dB steps) at a fixed *f*_p_, was used to determine the suppression criterion (see details in section of *Suppression Criterion*) for SFOAE STC recordings. For ease of comparison, the same *f*_p_ and *L*_*p*_ were adopted in SFOAE STCs and PTCs tests. *L*_p_s of 30 dB SPL and 50 dB SPL corresponding to low and moderate levels were used.

### Psychophysical tuning curves

PTC is constructed of different masker levels that subject just cannot hear the pure tone (complete masking) in a tracking paradigm as a function of masker frequency (*f*_m_) at a fixed *f*_p_ and *L*_p_. A repetition of the probe was 700-ms duration consisting 200-ms interval and 500-ms pure tone, and the rise/decay time of the pure tone was 20 ms. The number of repetitions was 350 for a total duration of 245 s. The masker was a 240-s narrowband noise and generated 5 s after the probe to enable subjects to recognize the target tone. The centre frequency of masker varied ± 1 octave relative to the *f*_p_ slowly from low to high (upward sweep) or high to low (downward sweep). Its frequency bandwidth was .2 times of the *f*_p_ and less than 320 Hz. The masker level (*L*_m_) was increased/decreased at a fixed rate of 2 dB/s when subjects can/cannot hear the probe tone with a maximum of 80 dB SPL. The raw PTC was a jagged curve for both upward and downward sweeps. Two-point average smoothing [30] was utilized to find the trend and estimate the tip frequency. Subjects were trained for 5 min prior to the data recording to familiar with the test paradigm.

### SFOAE STCs

SFOAE STC, obtained in an analogous way to PTC, is constructed for several critical *L*_s_s adjusted until the predefined suppression criterion is met as a function of *f*_s_ at the fixed *f*_*p*_ and *L*_*p*_. The *f*_s_ ranged from .5 *f*_*p*_ to 2.5 *f*_*p*_ at 1 kHz, but was limited to 1.75 *f*_*p*_ at 2 and 4 kHz because the *L*_s_ will saturate when *f*_s_ is near 2*f*_*p*_. SFOAE recording used six-section stimuli paradigm (see details in Fig. 2 of Gong et al. [30]) which was based on the two-tone suppression method of Brass et al. [20, 21]. The final result, a suppressed SFOAE, was the subtraction between the acoustic response to the probe alone and the probe in the presence of a suppressor tone.

**Fig. 2:**
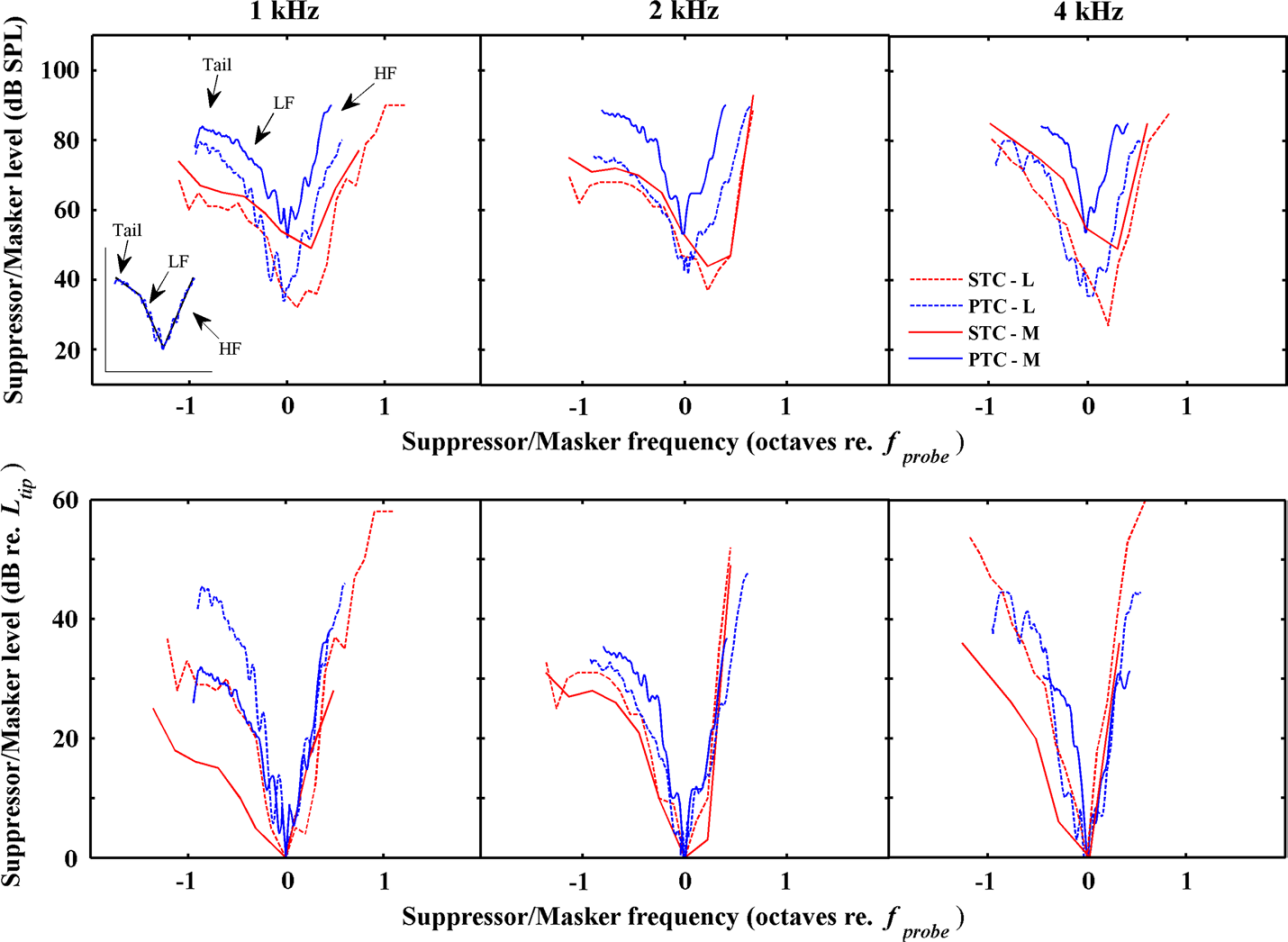
Individual raw data for SFOAE STCs and PTCs. The tuning curves are grouped in columns from left to right according to the *f*_p_s of tuning curves (centred at 1, 2, and 4 kHz respectively). SFOAE STCs: *red lines*; PTCs: *blue lines*. Low probe levels: *dotted lines*; moderate probe levels: *solid lines*

### Suppression criterion

In the detection of PTCs, we determined critical *L*_m_ based on subjects’ subjective judgement that they can or cannot hear the probe tone. While in the detection of SFOAE STCs, we utilized a predefined suppression criterion to determine the critical *L*_s_. The suppression criterion represented the SFOAE suppression extent relative to the total SFOAE. For example, the criterion of −6 dB corresponds to an amplitude decreased by a factor of 1/2 [28]. If the amplitude in SFOAE I/O function test still had enough signal to noise ratio (SNR ≥10 dB) when SFOAE suppressed 50 %, −6 dB will be chosen as a criterion. If not, the weaker criterion will be more appropriate. A criterion of −6 dB was chosen for all subjects in order to make the comparison under the same suppression condition.

### Data analysis

To calculate the characteristic parameters of SFOAE STCs and PTCs, their low-frequency sides (suppressor/masker frequency <*f*_p_, referred here as LF; suppressor/masker frequency, referred here as *f*_s,m_), high-frequency sides (*f*_s,m_ > *f*_p_, referred here as HF) and their low-frequency tails (*f*_s,m_ ≪ *f*_p_, with evident shallower slope than LF, referred here as tail) were fitted with regression lines (fitted lines, tail, LF and HF are pointed in the inserted figure of Fig. 2). The point adjacent the tail and LF side was defined as the boundary point which had the biggest slope difference on the tail and LF side. Characteristic parameters (*Q*_10_, slopes, frequency shift at the tip and level at the tip) of both SFOAE STCs and PTCs at different probe levels were calculated from the fitted curves. In order to assess the influence of *L*_p_ on the relationship between SFOAE STCs and PTCs, the ratio/difference was calculated between each parameter of the two methods at different *L*_p_s.

Means (Ms) and standard deviations (SDs) were provided first to compare the variation tendency of parameters for SFOAE STCs and PTCs at different *L*_p_s and *f*_p_s separately. Then data were analysed with nonparametric statistics (two-factor Scheirer-Ray-Hare test [37]) as most of them did not satisfy the homogeneity of variance requirements. In addition, the Pearson correlation coefficient was utilized to access the correlation between (1) two methods of estimating FS at both low and moderate *L*_p_s (correlation analysis was corrected in a way described in Bland et al. [38]); (2) repeated measures within one subject. *P* < .05 was considered statistically significant. All data analyses were conducted in SPSS 23 software (SPSS, Inc., Chicago, IL).

## Results

Figure 2, *top row*, illustrates an example of individual raw data for SFOAE STCs (Fig. 2, red lines) and PTCs (Fig. 2, blue lines) at low (exhibited as ‘L’ in all figure legends; Fig. 2, dotted lines) and moderate (exhibited as ‘M’ in all figure legends; Fig. 2, solid lines) probe levels at *f*_p_s centred at 1, 2, and 4 kHz (from left to right column). Both SFOAE STCs and PTCs had a similar V-shape with a tail at both low and moderate levels. At both low and moderate *L*_p_s, the *f*_tip_ shifted slightly higher than *f*_p_ for SFOAE STCs whereas the *f*_tip_ of PTCs coincided with *f*_p_. The *L*_s,m_ at the tip was larger at the moderate *L*_p_ than that at low *L*_p_ for both SFOAE STCs and PTCs. The level differences at the tip between SFOAE STCs and PTCs were larger with increasing *L*_p_. In order to show the sharpness of tuning more intuitively, we normalized the tuning curves to their tips (Fig. 2, *bottom row*). PTCs were tuned more sharply at the moderate *L*_p_ than that at low *L*_p_ at 2 and 4 kHz but broadly at 1 kHz. Nevertheless, the SFOAE STCs were tuned more broadly at the moderate *L*_p_ than that at low *L*_p_. For both low and moderate *L*_p_s, LF slopes for SFOAE STCs were shallower than those for PTCs but HF slopes were similar for SFOAE STCs compared with PTCs.

### Sharpness of tuning

**Fig. 3:**
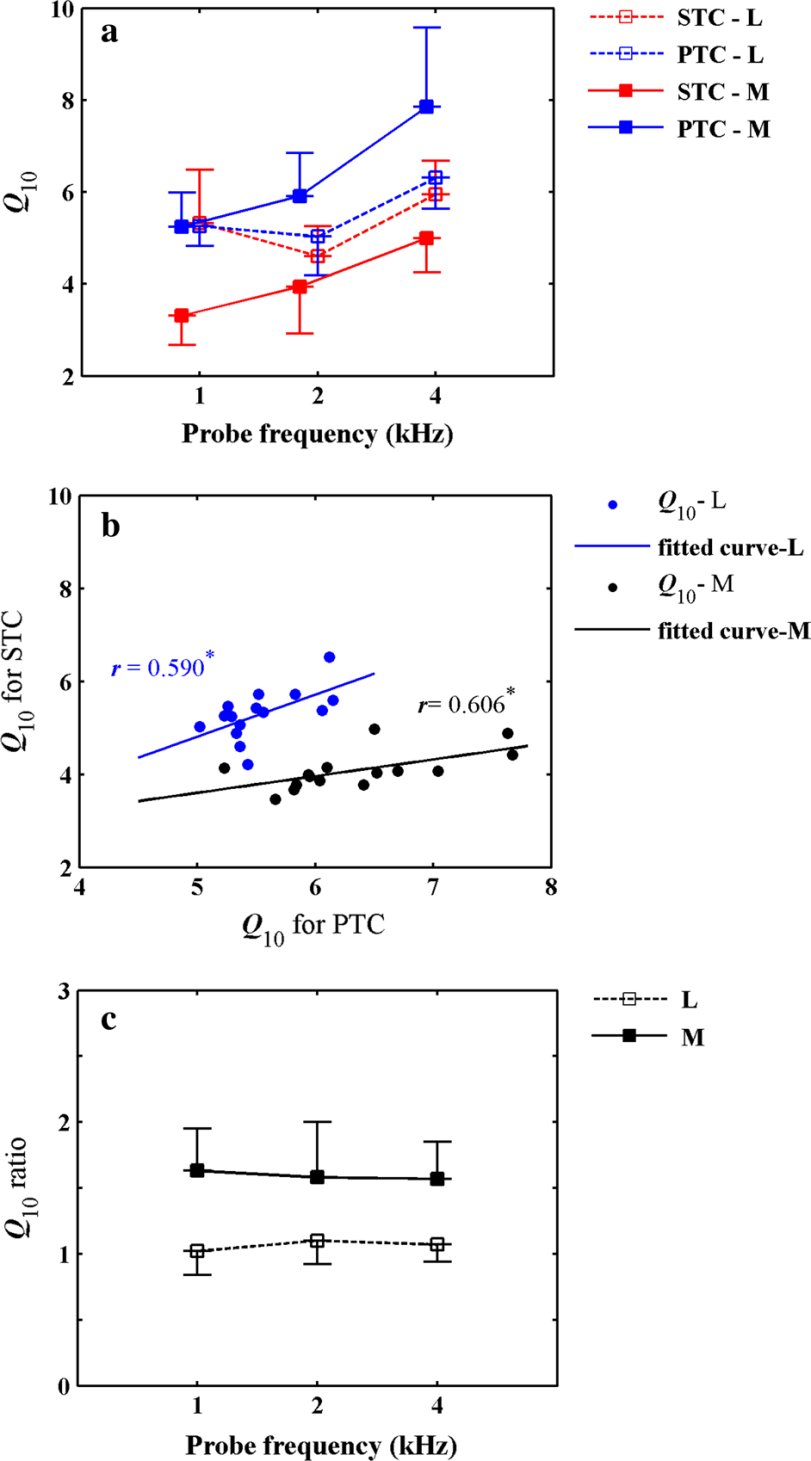
**a** Mean *Q*_10_ values of SFOAE STCs and PTCs as a function of *f*_p_. Mean *Q*_10_ values of SFOAE STCs (*red lines*) and PTCs (*blue lines*) as a function of *f*_p_ at *L*_p_s of 30 dB SPL (*dotted lines*) and 50 dB SPL (*solid lines*). *Error bars* denote ± 1 SD. **b** Correlation between *Q*_10_ values of SFOAE STCs and PTCs. Original data (*dots*) and linear fitted curves (*solid lines*) are presented at the *L*_p_s of 30 dB SPL (*blue*) and 50 dB SPL (*black*). Correlation coefficients are shown adjacent to the fitted curves. One *stars* indicate significant level at .05. **c** Mean *Q*_10_ ratios between SFOAE STCs and PTCs as a function of *f*_p_. Low probe levels: *dotted lines*; moderate probe levels: *solid lines*. *Error bars* denote ±1 SD

Conventionally, we adopt *Q*_10_ value to characterize the sharpness of tuning derived from SFOAE STCs and PTCs. It is a dimensionless quality factor relevant to FS, calculated as the ratio between the tip frequency of the tuning curve and the bandwidth at 10 dB above the tip (*Q*_10_ = *f*_tip_/BW10). Figure 3a illustrates mean *Q*_10_ values of SFOAE STCs and PTCs at both low and moderate probe levels as a function of *f*_p_. For SFOAE STCs, mean *Q*_10_ value at the moderate *L*_p_ was smaller than that at low *L*_p_. Whereas for PTCs, mean *Q*_10_ value at the moderate *L*_p_ was larger than that at low *L*_p_, at least at 2 and 4 kHz. Mean *Q*_10_ values of both SFOAE STCs and PTCs increased as a function of *f*_p_ at the moderate level but had a small notch at 2 kHz at the low *L*_p_. From Fig. 3b we can observe that, *Q*_10_ values of SFOAE STCs were significantly positively correlated with the *Q*_10_ values of PTCs at both low and moderate *L*_p_s (*L*: *r* = .590, *P* = .021; *M*: *r* = .606, *P* = .017).

*Q*_10_ ratios are *Q*_10_ values of PTCs divided by the *Q*_10_ values of SFOAE STCs for each subject which express the relationship of tuning between PTCs and SFOAE STCs. Figure 3c illustrates mean *Q*_10_ ratios between SFOAE STCs and PTCs as a function of *f*_p_. As shown in Fig. 3c, mean *Q*_10_ ratios were larger at the moderate *L*_p_ (*M* = 1.59, *SD* = .34) than that at low *L*_p_ (*M* = 1.06, *SD* = .17), which meant that PTCs were more sharply tuned than SFOAE STCs at the moderate *L*_p_. It was also shown that the mean *Q*_10_ ratios at the low *L*_p_ were constantly around 1, whilst the mean *Q*_10_ ratios at the moderate *L*_p_ were most distributed around 1.5, and they were independent of *f*_p_ at both *L*_p_s. Additionally, a smaller variability of *Q*_10_ ratios was observed at the low *L*_p_. Two-factor Scheirer-Ray-Hare test revealed that *L*_p_ had a significant effect on the *Q*_10_ ratios between SFOAE STCs and PTCs (*df* = 1, *H* = 12.4379, *P* = .0004) and *f*_p_ had no significant effect (*df* = 2, *H* = .0211, *P* = .9895). No interactions were found between the factors of level and frequency (*df* = 2, *H* = .3225, *P* = .8511).

### Slopes of tuning curves

The slopes of tails, low- and high- frequency sides are given by the quotient of the suppressor/masker level difference (Δ*L*_s,m_) and the normalized frequency difference (Δ*f*_s,m_/*f*_p_) with unit of *dB/octave*:

1 $$slopes\, = \,\frac{{\Updelta L_{s,m} }}{{\log_{2} ({{\varDelta f_{s,m} } \mathord{\left/ {\vphantom {{\Updelta f_{s,m} } {f_{p} }}} \right. \kern-0pt} {f_{p} }})}}$$

**Fig. 4:**
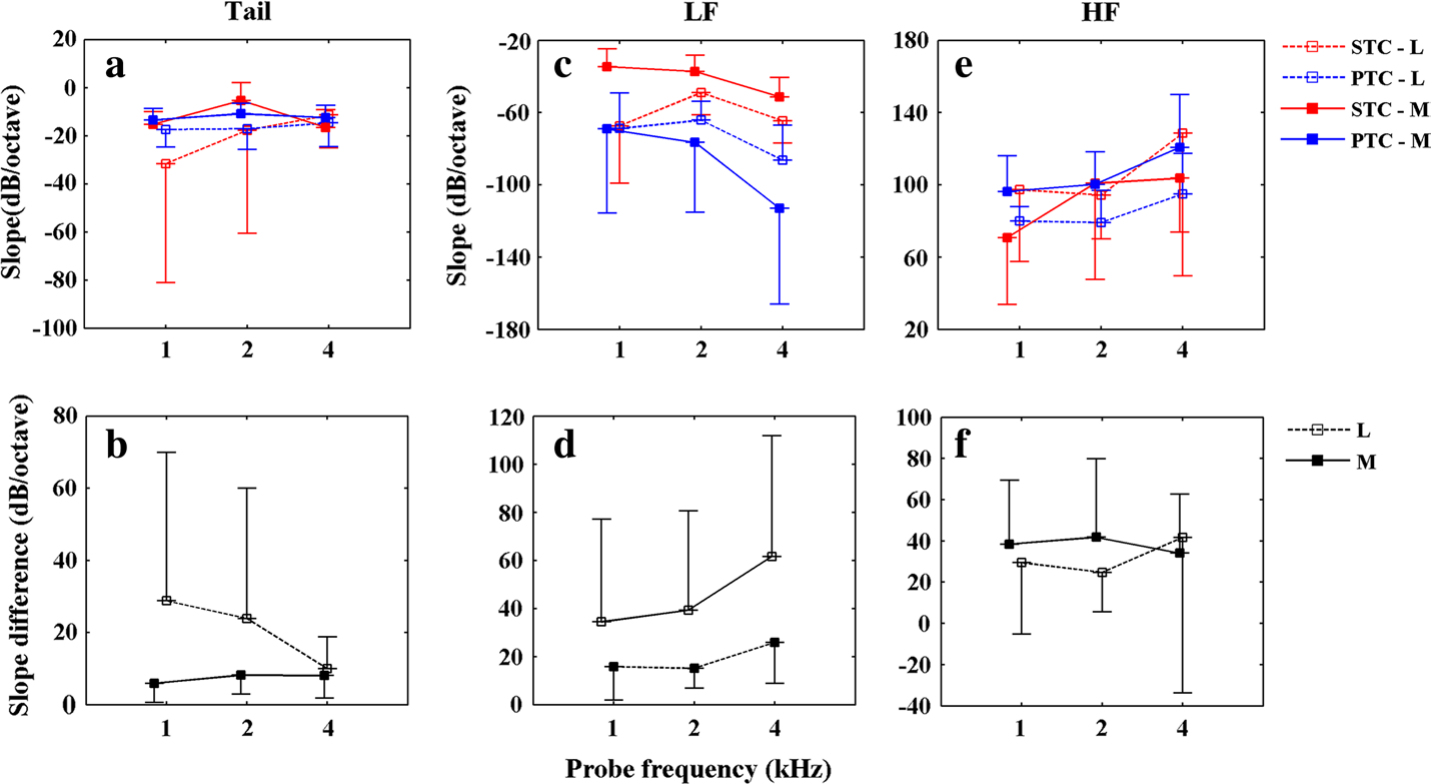
Mean values of slope and slope difference for SFOAE STCs and PTCs. Mean values of slopes (*top row*) and slope differences (*bottom row*) of tail, LF and HF (from *left *to* right* column) for SFOAE STCs (*red lines*) and PTCs (*blue lines*) as a function of *f*_p_ at *L*_p_s of 30 dB SPL (*dotted lines*) and 50 dB SPL (*solid lines*). *Error bars* denote ±1 SD

Slope of tuning curve describes the tuning characteristic on each frequency side, steeper slope indicates sharper tuning. In order to compare the difference between the slope of SFOAE STCs and PTCs at different probe levels, the slope difference using the absolute value for each subject is also calculated as the slope of PTCs minus the slope of SFOAE STCs. Figure 4 illustrates the mean values of slope and slope difference (absolute value) for tail, LF and HF between SFOAE STCs and PTCs at both low and moderate probe levels as a function of *f*_p_.

*Tail slopes* (Fig. 4a, b). The mean slopes of tails for SFOAE STCs and PTCs were smaller at the moderate *L*_p_ than that at low *L*_p_ except for the SFOAE STCs at 4 kHz. At low probe levels, mean tail slopes of SFOAE STCs and PTCs were decreasing as *f*_p_ increasing. At moderate probe levels, mean tail slopes of both SFOAE STCs and PTCs were getting smaller at *f*_p_ from 1 to 2 kHz, but larger at *f*_p_ from 2 to 4 kHz especially for SFOAE STCs. Although the mean tail slope difference (Fig. 4b) was larger at the low *L*_p_ (*M* = 20.88, *SD* = 32.32) than that at moderate *L*_p_ (*M* = 7.39, *SD* = 5.61), it did not reach significance. Two-factor Scheirer-Ray-Hare test revealed that neither *L*_p_ nor *f*_p_ had a significant effect on the tail-slope differences (absolute value) between SFOAE STCs and PTCs (*L*_p_: *df* = 1, *H* = 1.5209, *P* = .2175; *f*_p_: *df* = 2, *H* = .1590, *P* = .9236). No interactions were found between the factors of level and frequency (*df* = 2, *H* = .6875, *P* = .7091).

*LF slopes* (Fig. 4c, d). For SFOAE STCs, the mean LF slope was smaller at the moderate *L*_p_ than that at low *L*_p_. Whereas for PTCs, the mean LF slope was larger at the moderate *L*_p_ than at low *L*_p_. In addition, the variability for LF slopes of PTCs increased at higher *L*_p_. At low probe levels, mean LF slopes of both SFOAE STCs and PTCs were getting smaller at *f*_p_ from 1 to 2 kHz, but larger at *f*_p_ from 2 to 4 kHz. At moderate probe levels, mean LF slopes of both SFOAE STCs and PTCs were larger with increasing *f*_p_. The mean LF slope of PTCs was larger than SFOAE STCs at both low and moderate levels. Although the mean LF slope difference (Fig. 4d) was larger at the moderate *L*_p_ (*M* = 45.12, *SD* = 45.49) than that at low *L*_p_ (*M* = 18.93, *SD* = 14.15), it did not reach significance. Two-factor Scheirer-Ray-Hare test revealed that neither *L*_p_ nor *f*_p_ had a significant effect on the LF-slope differences (absolute value) between SFOAE STCs and PTCs (*L*_p_: *df* = 1, *H* = .9758, *P* = .3232; *f*_p_: *df* = 2, *H* = 1.5925, *P* = .4510), and no interactions were found between the factors of level and frequency (*df* = 2, *H* = .0449, *P* = .9778).

*HF slopes* (Fig. 4e, f). The mean HF slopes for PTCs were larger at higher *L*_p_. While for SFOAE STCs, mean HF slopes were smaller at higher *L*_p_ at 1 and 4 kHz but larger at 2 kHz. At low probe levels, mean HF slopes of both SFOAE STCs and PTCs were smaller at *f*_p_ from 1 to 2 kHz but larger at *f*_p_ from 2 to 4 kHz. At moderate probe levels, the mean HF slope of PTCs and SFOAE STCs were larger as *f*_p_ increasing. The mean HF slope difference (Fig. 4f) was larger at moderate *L*_p_ (*M* = 38.12, *SD* = 32.22) than that at low *L*_p_ (*M* = 31.98, *SD* = 48.61), which resembled the trends of LF slope differences. This agreed the observation that *Q*_10_ ratios were close to one at low probe levels. Two-factor Scheirer-Ray-Hare test revealed that neither *L*_p_ nor *f*_p_ had a significant effect on the HF-slope differences (absolute value) between SFOAE STCs and PTCs (*L*_p_: *df* = 1, *H* = .7174, *P* = .3970; *f*_p_: *df* = 2, *H* = .0921, *P* = .9550). No interactions were found between the factors of level and frequency (*df* = 2, *H* = .0291, *P* = .9856).

### Frequency shift at the tip

Frequency shift at the tip is given by the quotient of the frequency shift at the tip (relative to the probe) and *f*_p_, with unit of *%*:

2 $$Frequency \, shift \, of \, tip \, = \,\frac{{f_{tip} - f_{p} }}{{f_{p} }}$$

**Fig. 5:**
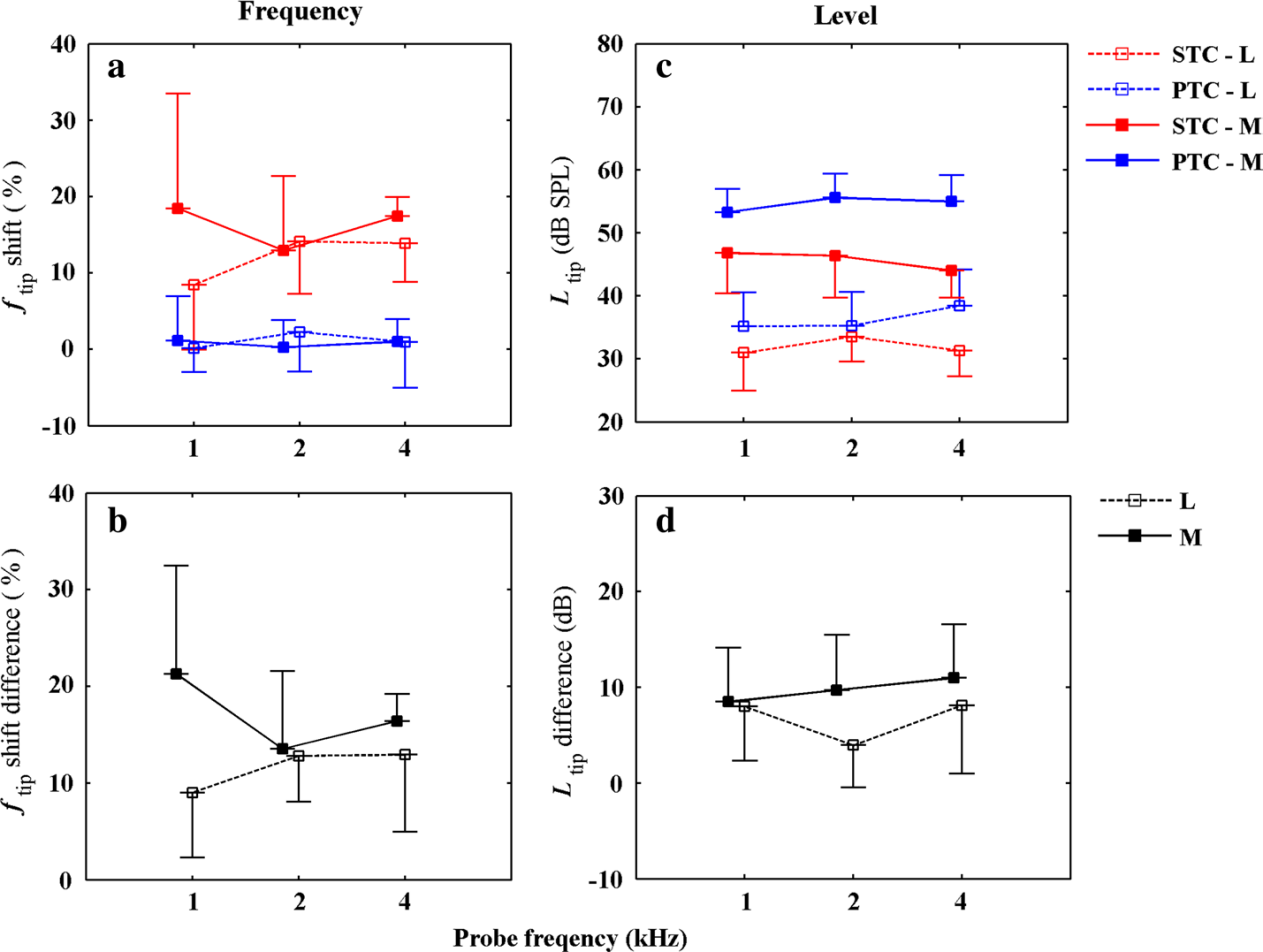
Mean values of *f*_tip_ shift/*L*_tip_ and *f*_tip_-shift/*L*_tip_ difference for SFOAE STCs and PTCs. Mean values of *f*_tip_ shift and *f*_tip_ shift difference (**a**, **b**) and *L*_tip_ and *L*_tip_ difference (**c**, **d**) for SFOAE STCs and PTCs as a function of *f*_p_ at the *L*_p_s of 30 dB SPL (*dotted lines*) and 50 dB SPL (*solid lines*). *Red lines*: SFOAE STCs; *blue lines*: PTCs. *Error bars* denote ±1 SD

In order to compare the difference between the *f*_tip_ shift of SFOAE STCs and PTCs at different probe levels, the *f*_tip_ shift difference using the absolute value for each subject is calculated as the *f*_tip_ shift of PTCs minus SFOAE STCs’. Mean values of frequency shift and frequency shift difference at the tip for SFOAE STCs and PTCs at both low and moderate probe levels as a function of *f*_p_ are illustrated in Fig. 5a and b respectively. The SFOAE STCs shift was higher at the moderate *L*_p_ than that at lower *L*_p_ except for 2 kHz, while the tips of PTCs always coincided with *f*_p_ independent of *f*_p_ and *L*_p_ (Fig. 5a). The mean *f*_tip_ shift difference at the moderate *L*_p_ was larger than that at low *L*_p_ (Fig. 5b). Two-factor Scheirer-Ray-Hare test revealed that neither *L*_p_ nor *f*_p_ had a significant effect on the frequency shift differences (absolute value) between SFOAE STCs and PTCs (*L*_p_: *df* = 1, *H* = 3.0495, *P* = .0808; *f*_p_: *df* = 2, *H* = .3401, *P* = .8436), and no interactions were found between the factors of level and frequency (*df* = 2, *H* = 1.4371, *P* = .4875).

### Level at the tip

In order to compare the difference between the level at the tip (*L*_tip_) of SFOAE STCs and PTCs at different probe levels, the *L*_tip_ difference using the absolute value for each subject is calculated as the *L*_tip_ of PTCs minus SFOAE STCs’. Mean values of level and level difference at the tip for SFOAE STCs and PTCs at both low and moderate probe levels as a function of *f*_p_ are illustrated in Fig. 5c, d respectively. Both the mean suppressor/masker levels at the tip for SFOAE STCs and PTCs were larger with increasing *L*_p_ and were independent of *f*_p_ (Fig. 5c). As *L*_p_ increasing, the mean *L*_tip_ difference increased as well (Fig. 5d). Two-factor Scheirer-Ray-Hare test revealed that neither *L*_p_ nor *f*_p_ had a significant effect on the *L*_tip_ differences (absolute value) between SFOAE STCs and PTCs (*L*_p_: *df* = 1, *H* = 1.7440, *P* = .1829; *f*_p_: *df* = 2, *H* = .7835, *P* = .6759), and no interactions were found between the factors of level and frequency (*df* = 2, *H* = .7892, *P* = .6739).

### Repeatability

**Fig. 6:**
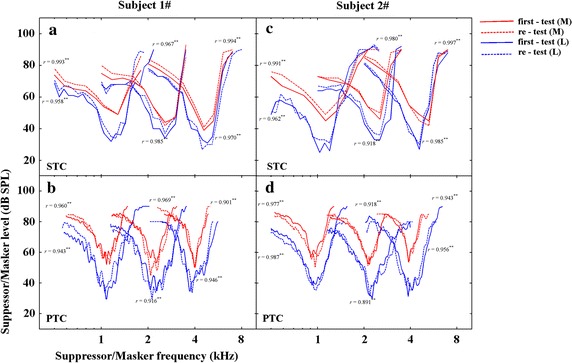
First and repeated measurements of SFOAE STCs and PTCs in two subjects. First measurements (*solid lines*) and repetition (*dotted lines*) for SFOAE STCs (*top row*) and PTCs (*bottom row*) at different *f*_p_s and *L*_p_s. Low probe levels: *blue lines*; moderate probe levels: *Red lines*. Correlation coefficients are shown adjacent to the corresponding tuning curves. *Double stars* indicate significant level at .01

Pairs of SFOAE STCs and PTCs in two subjects for frequencies centred at 1, 2, and 4 kHz were first measured (Fig. 6, solid lines) and re-measured (Fig. 6, dotted lines) after 26 days at both low (Fig. 6, blue lines) and moderate (Fig. 6, red lines) probe levels to verify the test-retest reliability. For SFOAE STCs, two curves of the original and the repetition across two subjects were correlated significantly (*P* < .001, the correlation coefficients are indicated adjacent to the corresponding tuning curves in Fig. 6). The difference between the repetition and the first measurement at corresponding *f*_p_ was calculated, with a grand average of .9 dB. For PTCs, two curves of the first and the repetition across two subjects were correlated significantly at the same condition (*P* < .001, the correlation coefficients are indicated adjacent to the corresponding tuning curves in Fig. 6). The grand average of difference between the repetition and the first measurement at corresponding *f*_p_ was −.6 dB. Our results were in consistence with the previous test-retest data for SFOAE STCs reported by Keefe et al. [28] and Charaziak et al. [29] with the grand average value of .6 and −.8 dB respectively. Consequently, the measurements of SFOAE STCs and PTCs for an individual ear were repeatable in our study within an acceptable variation.

## Discussion

The present study compared the shape and tuning of SFOAE STCs and PTCs at both low and moderate *L*_p_s in 15 normal-hearing subjects at frequencies around 1, 2, and 4 kHz. The trends for changes in the various tuning parameters are shown in Table 1 across the probe frequencies and levels. The results demonstrated that *L*_p_ had a significant effect on the sharpness of tuning between the two methods. The tuning of SFOAE STCs was positively correlated with the tuning of PTCs at both low and moderate *L*_p_s. Sharpness of tuning for SFOAE STCs was similar to the PTCs’ at lower probe levels. However, as probe level grows, SFOAE STCs were tuned broadly whereas PTCs were tuned sharply.

**Table 1:** The trends for changes in all parameters of SFOAE STCs and PTCs

Items			*L*_*P*_ (ranging from low to moderate)			*F*_*p*_ (ranging from 1 to 2 kH/from 2 to 4 kHz)	
			*F*_*p*_ = 1 kHz	*F*_*p*_ = 2 kHz	*F*_*p*_ = 4 kHz	Low	Moderate
Tuning	*Q*_10_	SFOAE STCs	−	−	−	−/+	+/+
		PTCs	≈	+	+	−/+	+/+
		Ratio	+	+	+	+/≈	≈/≈
Slopes	Tail	SFOAE STCs	−	−	+	−/−	−/+
		PTCs	−	−	−	≈/−	−/+
		Difference	−	−	−	−/−	+/≈
	LF	SFOAE STCs	−	−	−	−/+	+/+
		PTCs	≈	+	+	−/+	+/+
		Difference	+	+	+	+/+	+/+
	HF	SFOAE STCs	−	+	−	−/+	+/+
		PTCs	+	+	+	−/+	+/+
		Difference	+	+	−	−/+	+/−
Shift at the tip	Frequency	SFOAE STCs	+	−	+	+/≈	−/+
		PTCs	+	−	≈	+/−	−/+
		Difference	+	+	+	+/≈	−/+
	Level	SFOAE STCs	+	+	+	+/−	−/−
		PTCs	+	+	+	≈/+	+/≈
		Difference	+	+	+	−/+	+/+

“+” represents increasing, “−” represents decreasing, “≈” represents little variation (variation ≤5 %). The variation range for *f*_*p*_ is divided into two sections which is ranging from 1 to 2 kHz and from 2 to 4 kHz, respectively

### Tuning relationship between SFOAE STCs and PTCs

Our results revealed that the asymmetry shape of SFOAE STCs and PTCs were existing at both low and moderate *L*_p_s (Fig. 2), which agreed with other observations of SFOAE STCs [28, 29] and PTCs [13]. We also observed that the tuning of SFOAE STCs was positively correlated with the tuning of PTCs at both low and moderate *L*_p_s (Fig. 3b). It reveals that shaper tuning of SFOAE STCs will predict sharper tuning of PTCs at both low and moderate *L*_p_s. Although the correlation between the *Q*_10_ values of two methods seems better at higher probe levels from the aspect of correlation coefficient (*r* = .590 for low probe levels, *r* = .606 for moderate probe levels), the *Q*_10_ values of PTCs were larger than SFOAE STCs at the moderate *L*_p_ and they were not equivalent (Table 1; Fig. 3c). At lower *L*_p_, the SFOAE STCs indicated similar tuning to PTCs which was consistent with previous studies [28, 29]. The tuning similarity at lower levels suggests that PTCs are shaped by cochlear mechanics to a large extent at lower *L*_p_. Additionally, *Q*_10_ ratio between SFOAE STCs and PTCs was also independent of *f*_p_ at different *L*_p_s. Moreover, larger variability of the *Q*_10_ ratio at the moderate *L*_p_ compared with the low *L*_p_ was observed in our study (Fig. 3c). It indicates that, at lower *L*_p_s, SFOAE STCs appear more reliable to replace PTCs for FS estimation from the aspect of individual. As probe level grows, SFOAE STCs were tuned broadly whereas PTCs were tuned sharply. It may partially due to the involvement of higher auditory processing stages for PTCs. Although PTCs are shaped by cochlear mechanics to a large extent, it also involves signal processing in the central auditory system.

### The opposite effect of probe level on SFOAE STC and PTC tuning

Our results suggested that the SFOAE STCs represented higher acuity at low *L*_p_ than that at moderate *L*_p_, which was in consistent with the observation in humans at 4 kHz [29] and in chinchillas [27]. The FS estimate based on SFOAEs group delay was also observed decreasing tuning with increasing *L*_p_ ranging from 40 to 70 dB SPL in an approximately frequency-independent manner in humans (see Figure 8 of Schairer et al. [31]). Nonlinearity of BM mechanics [34] is likely responsible for the broader tuning of SFOAE STCs at higher *L*_p_ in our study as the motion of BM may exhibit less sensitive response to the higher probe level compared with low probe level and lead to poor tuning. However, little variation of *Q*_ERB_ as *L*_p_ increasing was observed in human SFOAE STCs as reported by Keefe et al. (see Figure 9 of [28]). Additionally, the sharpness of tuning for SFOAE STCs at 1 kHz reported by Charaziak et al. (see Figure 9 of [29]) was not in accordance with our finding at 1 kHz. This difference may results from the different probe levels (SL vs. SPL) and different definitions for suppression criterions (dB SPL vs. dB) as well as individual differences.

*Q*_10_ values of PTCs in our study suggested increasing tendency with the *L*_*p*_ changing from 30 to 50 dB SPL except for *f*_p_ at 1 kHz (Table 1; Fig. 3a), which was consistent with the simultaneous-masking PTC data (*L*_p_ in the same variation range with our study) of Stelmachowicz and Jesteadt who used narrow-band noise as a masker [16]. A similar tendency in changes in sharpness of tuning was also observed in the investigation of Oxenham et al. who adopted notched-noise as a masker even though they attributed this tendency to the individual differences in FS (see Figure 7 in Oxenham et al. [39]). In contrast, the forward-masking PTC was broader at higher probe levels [15]. The increasing *Q*_10_ at higher *L*_p_ in our study can be explained by the observation of Nelson et al. [40] that the presence of the combination-tone [41, 42] and other off-frequency listening cues essentially steepened the LF slopes at high probe levels at 2 and 4 kHz, resulting in shaper estimates of tuning. Nelson et al. [40] also observed that PTCs at higher *L*_p_ were influenced very little by the combination-tone at the low probe frequency (1 kHz). Thus we suspect that the little variation of *Q*_10_ for PTCs at 1 kHz with increasing *L*_p_ in our study may be related to the combination tones. Additionally, individual differences are partially contributed to the little variation of tuning at 1 kHz (e.g., individual PTC is broaden at 1 kHz at the higher probe levels in Fig. 2). The similar finding that individual PTC of *f*_p_ at 1 kHz represented broad tuning at the higher *L*_p_ was also observed in other investigations [13, 14].

### Slopes of tuning curves

Our results suggested that slope difference of tuning curve was larger at higher probe levels with the exception of tail slope (Table 1; Fig. 4b, d and f). Flatten LF slope at higher probe levels for SFOAE STCs was observed in our study (Table 1; Fig. 4c), whereas Charaziak et al. reported that the LF slope was not changing significantly with the increasing probe level [29]. HF slopes of SFOAE STCs were smaller at the higher *L*_p_ at 1 and 4 kHz but larger at 2 kHz (Table 1; Fig. 4e). It appears a non-monotonic function and little variation with increasing *L*_p_.

For PTCs, tail slope decreased slightly with increasing *L*_p_ which agreed with the finding of Nelson et al. [15]. Larger LF slope of PTCs at the higher *L*_P_ at 2 and 4 kHz in our results was consistent with the observation of Stelmachowicz and Jesteadt (*L*_p_ varying from 30 to 50 dB SPL) [16]. In contrast, some investigators reported that the LF slope was flatten at the higher *L*_P_ [14, 15]. LF slopes of PTCs at 1 kHz revealed little variation with increasing *L*_p_ in the present study (Table 1; Fig. 4c), while the previous reported data indicated that LF slopes of PTCs at 1 kHz decreased with increasing *L*_p_ [13, 44]. The increasing variability of LF slope for PTCs at higher probe levels in our study (Fig. 4c) was also observed by Stelmachowicz and Jesteadt [16]. It is probably related to the detection of combination tones or combination band cues [40]. The HF slopes of PTCs were larger at the higher *L*_p_ (Table 1; Fig. 4e). Although this finding was consistent with the previous study [16], others indicated that the HF slope was independent of *L*_p_ [13, 15].

### Frequency shift at the tip

Our results suggested that the *L*_p_ had no significant effect on the *f*_tip_ shift difference between the two tuning curves. The *f*_tip_ of SFOAE STCs was always shifted 1.1 ~ 1.2 *f*_*p*_ regardless of *L*_p_, which agreed with other SFOAE studies [21, 28]. Other types of otoacousitc emission (OAE) STCs were also observed that they were tuned to a frequency higher than the probe frequency [44, 45]. It complied with the observation of the characteristic of two-tone suppression mechanism in BM [46] and auditory nerve fibres [47] that the maximum sensitive frequency was slightly higher than *f*_p_. Thus the two-tone suppression may be contribute to the tip shift of SFOAE STCs. The *f*_tip_ of SFOAE STC shifted higher relative to the *f*_p_ with increasing *L*_p_ (the mean frequency shifts at the tip were 12.16 % at the low *L*_p_ and 16.26 % at the moderate *L*_p_). This increasing size of *f*_tip_ shift at the higher *L*_p_ observed in our study was consistent with Charaziak et al. [30]. It implicates that the two-tone suppression mechanism may be affected by the probe level to a large extent. In addition, the shift at SFOAE STCs’ tip may be presumably related to SFOAE being generated slightly basal to the characteristic place of the probe [25, 29, 48]. PTCs in our study always coincided with *f*_*p*_ regardless of *L*_*p*_, which was in agreement with the previous study [43]. It supports the phenomenon that PTCs have a tip close to the signal frequency in normal-hearing subjects. However, Carney et al. [9] found that the *f*_tip_ shifted toward slightly lower frequencies as probe level increasing. The inconsistence in that literature may due to the methodological difference for determining a PTC.

### Level at the tip

Our results suggested that the *L*_p_ had no significant effect on the *L*_tip_ difference between SFOAE STCs and PTCs. At both low and moderate *L*_p_s, the suppressor/masker level required at a characteristic frequency was larger for PTCs than SFOAE STCs. This difference is likely due to the different suppression/masking criterion between SFOAE STC (50 % suppressed) and PTC (fully masking). Additionally, the suppressor/masker levels of tip for both SFOAE STCs and PTCs were increasing with *L*_p_ (Table 1; Fig. 5c), which was consistent with other observations of SFOAE STCs [28] and PTCs [13, 43].

### Application and limitation

At low probe levels, the *Q*_10_ ratio was close to one for all tested frequency suggesting that the PTCs and SFOAE STCs were equivalent measures of FS. As far as the method for data collected in our study, SFOAE STCs take longer to record compared with PTCs. Even though SFOAE STCs can evaluate FS objectively, time-inefficiency of the measurements is one limitation for clinical use. Previous studies reported that SFOAE STCs can be measured reliably within 10–15 min [27, 29], but it still lasts longer than PTCs. Therefore, it is crucial to investigate a fast method for SFOAE STCs in the further study. The combination tones and off-frequency listening detection may emerge for PTCs at higher probe levels in normal-hearing listeners whereas it does not exist in hearing-impaired listeners [40]. Consequently, the opposite effect of *L*_p_ on the SFOAE STCs and PTCs tuning indicates that SFOAE STCs are more suitable for FS evaluation compared with PTCs in normal-hearing listeners for whom higher probe levels are necessary. Despite of difficulties for SFOAE STCs in obtaining satisfactory SNR at the higher *L*_p_ due to the on-band noise problem observed in the study of Charaziak et al. [49], Charaziak et al. [49] also predicted that tuning evaluation method based on SFOAE with an alternative method (e.g., amplitude-modulated SFOAE [50]) may be more reliable at higher probe levels. Thus, the reliability of SFOAE STCs or other FS measurement based on SFOAEs in abnormal-hearing listeners is required for further investigation. Additionally, the data in the present study may provide a basis for comparison between normal-hearing listeners and hearing-impaired listeners for whom higher-level probes are generally necessary.

## Conclusions

In this study, the effect of probe level on the relationship of tuning and shape between SFOAE STCs and PTCs in normal-hearing subjects was compared. The results showed that the probe level had a significant effect on the tuning relationship between the two methods. At low probe levels, SFOAE STC seems a good alternative measurement of PTC for FS assessment. However, at moderate probe levels, SFOAE STCs were broader whereas PTCs were sharper and they were not equivalent. SFOAE STCs may represent cochlear FS better than PTCs because they are not biased by higher levels auditory processing.

## Abbreviations

FS: frequency selectivity
HF: high-frequency side
LF: low-frequency side
PTC: psychophysical tuning curves
SD: standard deviation
SFOAE: stimulus frequency otoacoustic emission
SPL: sound pressure level
STC: suppression tuning curve
